# Association between traditional cardiovascular risk factors and mortality in the oldest old: untangling the role of frailty

**DOI:** 10.1186/s12877-017-0626-x

**Published:** 2017-10-12

**Authors:** Bert Vaes, David Depoortere, Gijs Van Pottelbergh, Catharina Matheï, Joana Neto, Jan Degryse

**Affiliations:** 1 Department of Public Health and Primary Care, Universiteit Leuven (KU Leuven), Leuven, Belgium; 2 Institute of Health and Society, Université catholique de Louvain (UCL), Brussels, Belgium

**Keywords:** Cardiovascular risk prediction, Hypertension, Cholesterol, Frailty, Mortality

## Abstract

**Background:**

To date, there is no consensus regarding cardiovascular risk management in the very old. Studies have shown that the relationship between traditional cardiovascular risk factors and mortality is null or even inverted within this age group. This relationship could be modified by the presence of frailty. This study was performed to examine the effect of frailty on the association between cardiovascular risk factors and mortality in the oldest old.

**Methods:**

The BELFRAIL study is a prospective, observational, population-based cohort study of 567 subjects aged 80 years and older. Data on cardiovascular risk factors were recorded. Frailty was assessed using three different models: the Groningen Frailty Indicator, Fried and Puts models. Participants were considered robust if they were ‘not frail’ according to all three models, and frail if they met the frailty criteria for one of the three models. The follow-up data on mortality and cause of death were registered.

**Results:**

No cardiovascular risk factor was associated with mortality in subjects with and without cardiovascular disease. The presence of frailty was a strong risk factor for mortality [HR: 2.5, 95%CI: (1.9–3.2) for all-cause mortality; HR: 2.2, 95%CI: (1.4–3.4) for cardiovascular mortality]. In robust patients, a history of cardiovascular disease increased the risk for mortality [HR: 1.7, 95%CI: (1.1–2.5) for all-cause mortality; HR: 2.2, 95%CI: (1.2–3.9) for cardiovascular mortality]. In frail patients, there was no association between any of the traditional risk factors and mortality.

**Conclusions:**

Traditional cardiovascular risk factors were not associated with mortality in very old subjects. Frailty was shown to be a strong risk factor for mortality in this age group. However, frailty could not be used to identify additional subjects who might benefit more from cardiovascular risk management.

**Electronic supplementary material:**

The online version of this article (10.1186/s12877-017-0626-x) contains supplementary material, which is available to authorized users.

## Background

In the ageing Western society, cardiovascular disease is highly prevalent, is a major cause of morbidity, disability and mortality and is still the leading contributor to overall burden of disease in older people [1]. In 2015 there were 11.3 million new cases of cardiovascular disease and more than 85 million people already living with cardiovascular disease in Europe [2]. Cardiovascular disease accounts for 53% (2.6 million) of all deaths in people aged 75 and older in Europe [3]. This places a heavy burden on health care systems. Therefore, cardiovascular prevention, both primary and secondary, remains a priority.

While there is an abundance of evidence for the importance of adequate cardiovascular risk management even at an older age [4, 5], more studies have emerged implying that when analysing the oldest age groups (aged 80 years and older), the predictive value of classical cardiovascular risk factors, such as hypertension or hypercholesterolaemia, is lost or even inverted [6, 7]. This might be related to the oldest old being a very heterogeneous population, ranging from an active independent community-dwelling elder to a bedridden geriatric patient. On the one hand, cardiovascular disease is associated with an increased likelihood of frail health [8]. But on the other hand, the impact of frailty could progressively dominate the prognosis, and subsequently diminish the predictive value of classic cardiovascular risk factors.

Consequently, identifying older individuals who are likely (not) to benefit from cardiovascular risk management is challenging. Several recent studies have advocated categorizing individuals based on biological instead of chronological age in order to facilitate personalized cardiovascular risk management for the ageing population, that is, using a patient-based approach instead of a disease-based approach [9].

Markers of frailty, such as gait speed, underlying comorbidities and polypharmacy, or a combined measure such as a frailty index, could be used to understand the complex relation between the classic cardiovascular risk factors and risk of clinical outcomes [9–11]. Therefore, this study was performed in order to determine the association between classic cardiovascular risk factors and all-cause and cardiovascular mortality according to the presence of frailty in a large prospective cohort of patients aged 80 years and older.

## Methods

### Study population

This study is embedded within the BELFRAIL study, a prospective, observational, population-based cohort study of subjects aged 80 years and older in Belgium. All the participants in the study gave written informed consent, and the Biomedical Ethics Committee of the Medical School of the Université catholique de Louvain (UCL) of Brussels approved the study. The study design, methods and characteristics of the cohort have been published in detail elsewhere [12]. Briefly, 29 general practitioner (GP) centres included 567 subjects between November 2, 2008 and September 15, 2009. Only three exclusion criteria were used: dementia [defined as having a known mini mental state examination (MMSE) score < 15/30], palliative care, and presence of a medical emergency. Patients were questioned and examined by both a GP and a clinical research assistant (CRA). The GP recorded social situation, medical history and medication, and conducted a thorough clinical examination. The CRA performed an extensive examination containing performance testing, several questionnaires, and technical examinations.

### Frailty

Various frailty models exist based on various conceptual and operational definitions. Currently, all frailty instruments could be divided into self-reported and performance-based ones.

The Groningen Frailty Indicator (GFI) [13] is a self-reported frailty instrument based on a 15-item questionnaire focusing on the core domains of functioning. The CRA assessed the GFI in all subjects. A person was considered frail when six or more items were present.

There are two widespread performance-based instruments for measuring frailty in older adults: the phenotype frailty model and frailty index of cumulative deficits. The frailty phenotype model or Fried model is closely linked to sarcopenia and defines frailty as a biological syndrome of decreased reserve and resistance to stressors that results from cumulative declines across multiple physiological systems [14]. This model consists of 5 items: unintentional weight loss (as reported by the general practitioner), weakness [measured grip strength (using a Jamar Plus digital hand-held dynamometer) in the lowest quintile], poor endurance/exhaustion (self-report of exhaustion), slowness (slowest quintile in a test of timed walking speed), and sex-adjusted low physical activity level [LASA (Longitudinal Aging Study Amsterdam) Physical Activity Questionnaire (LAPAQ) score in the lowest quintile)]. Individuals with three or more criteria were considered frail.

The cumulative deficit approach was developed based on the concept of the number of health “deficits” that are manifested in an individual. The Puts model [15] comprises nine frailty markers: low body weight (BMI <23 kg/m^2^), low FEV1 (lowest sex-adjusted quintile of forced expiratory volume in 1 s), poor cognition (MMSE <24), vision problems (asking the respondent are you able to recognize someone’s face at a distance of 4 m), hearing problems (asking the respondent are you able to follow a conversation with one and four persons), incontinence (asking the respondent whether he or she lost urine unintentionally), low sense of mastery [lowest quintile of sense of coherence (SOC-13)], depressive symptoms [15-item Geriatric Depression Scale (GDS-15) >5], and physical activity [LASA (Longitudinal Aging Study Amsterdam) Physical Activity Questionnaire (LAPAQ) score in the lowest quintile]. Each health ‘deficit’ such as hearing impairment contributes cumulatively to an increased risk of functional decline and death, although they do not each pose an obvious or imminent threat of mortality. Frailty was defined as the presence of three or more frailty markers [15].

In our current study, the participants were considered ‘robust’ if they were ‘not frail’ according to all three models, and participants were considered ‘frail’ if they met the frailty criteria for one of the three aforementioned models. The three different frailty models were all designed to cover a different latent construct of frailty. This approach was thus mainly used to identify ‘certainly robust’ subjects and identifying ‘possibly frail’ patients.

### Cardiovascular risk factors

The GP was asked to report the smoking status of the patient (no versus current or prior) and presence of hypertension, diabetes and cardiovascular disease. The presence of cardiovascular disease was classified as either minor (history of angina pectoris, transient ischaemic attack (TIA), peripheral arterial disease or episode of decompensated heart failure) or major (history of myocardial infarction {according to the GP or positive electrocardiogram [ECG] [Minnesota Code 1–1 or 1–2 (excluding 1–2-8)]**}**. stroke, percutaneous transluminal coronary angioplasty (PTCA) or stent, coronary surgery or arterial surgery) [16]. The GP measured the systolic and diastolic blood pressure in the sitting position on both arms; the measurement was repeated after 2 min. For the current study, only the highest readings (left or right) of the second measurement were used. Three categories of systolic blood pressure (<140 mmHg, 140–160 mmHg and ≥160 mmHg) and three categories of diastolic blood pressure were used (<70 mmHg, 70–90 mmHg and ≥90 mmHg). The CRA calculated the body mass index (BMI) based on a standardized measurement of weight and height.

A blood sample was collected in the morning after fasting, and serum samples were stored frozen at −80 °C until analysis. Total cholesterol, high-density lipoprotein cholesterol (HDL-C), low-density lipoprotein cholesterol (LDL-C) and the serum concentration of creatinine were measured using the UniCel® DxC 800 Synchron (Beckman-Coulter, Brea, CA, USA). Tertiles of total cholesterol and HDL-C were made. The glomerular filtration rate was estimated (eGFR) using the Modification of Diet in Renal Disease (MDRD) formula.

Data on relevant antihypertensive medication, including diuretics, B-blockers, calcium antagonists, angiotensin-converting enzyme inhibitors (ACE-I), angiotensin II receptor blockers (ARB) and potassium sparing agents, were recorded. Furthermore, the presence of lipid-modifying agents was also registered.

### Outcomes

Three detailed follow-up questionnaires were filled in by the participating GPs after 1.4 ± 0.3 years [mean ± standard deviation (SD)], after 3.0 ± 0.3 years and after 5.1 ± 0.3 years. These questionnaires included questions on mortality and cause of death. The causes of death were divided into cardiovascular and non-cardiovascular causes according to the GPs’ assessment and subsequent review by 2 independent researchers blinded to all clinical data.

### Data analysis

Continuous variables were presented as the mean and SD. Categorical variables were presented as numbers and frequencies. Comparisons between different categories of subjects were performed using the Chi^2^ test for categorical variables or Student’s t test for continuous variables. Survival curves were computed using the Kaplan-Meier method and were compared using the log-rank Chi^2^ squared test. Different strata based on the presence of frailty and tertiles of total cholesterol and HDL-C and predefined categories of systolic and diastolic blood pressure were made. Determination of the factors independently associated with outcomes was performed using Cox’s proportional hazards survival analysis. This analysis was performed on the total population, in subjects with and without a history of cardiovascular disease and in frail and robust subjects. All individual risk factors were adjusted for age, gender and level of education. Furthermore, all individual cardiovascular risk factors with *P* < 0.10 were proposed for inclusion into a multivariable Cox’s proportional hazards survival model. In order to avoid multicollinearity, the correlation coefficients between all covariates were calculated. In the case of multicollinearity (*r*-value >0.80), only one of the two covariables was considered in the multivariable model. The data analysis was performed using SPSS 20.0 for Windows (SPSS Inc., Chicago, IL, USA).

## Results

In total, 566 patients (99.8%) were included for the present study. A history of cardiovascular disease was present in 280 subjects (49.5%): a history of minor cardiovascular disease was present in 191/280 subjects (68.2%), and a history of major cardiovascular disease was present in 198/280 subjects (70.7%). At least one of the three frailty models was assessed in 559 patients: 123/559 subjects (22.0%) were frail according to the GFI, 112/543 (20.6%) were frail according to the Puts model, and 39/532 (7.3%) were frail according to the Fried criteria. The agreement between the three frailty models was low. The kappa statistics value for agreement between the GFI and the Puts model, the GFI and the Fried model and the Puts model and the Fried model were 0.41 (*P* < 0.001), 0.29 (*P* < 0.001) and 0.34 (*P* < 0.001) respectively. In total, 361/541 patients (66.7%) were robust according to all three models, and 180/541 patients (33.3%) were frail according to one of the three models. Although 18/559 subjects were considered to be robust according to one or two frailty models, at least one frailty assessment that could have shown the presence of frailty was missing. Therefore, these 18 subjects were excluded from further analyses using the presence of frailty. In total 24/180 were frail according to all three models, 46/180 according to two models and 110/180 according to only one model.

Table 1 gives an overview of the characteristics of the study population according to the presence of frailty. Frail subjects were more likely to be older, were more likely to be female and were less likely to be higher educated. A history of either minor or major cardiovascular disease was more often observed in the frail subjects. There was no significant difference between both groups regarding the presence of traditional cardiovascular risk factors. Frail persons had a lower eGFR than robust persons. The prescription of antihypertensive medication or cholesterol-lowering medication was not significantly different between frail and robust persons.

**Table 1:** Characteristics of BELFRAIL participants stratified by the presence of frailty (*n* = 541)

	Robust (*n* = 361)	Frail (*n* = 180)	*P* value
Age, mean (SD), y	84 ± 3.4	86 ± 3.9	<0.001
Male, %	155 (43)	47 (26)	<0.001
<High school education, %	120 (33)	80 (45)	0.009
Current/prior smoking, %	116 (32)	53 (30)	0.54
BMI, mean (SD), kg/m^2^	28 ± 4.2	27 ± 6.0	0.60
Total cholesterol, mean (SD), mg/dL	200 ± 45	203 ± 44	0.53
HDL-C, mean (SD), mg/dL	56 ± 15	54 ± 16	0.26
LDL-C, mean (SD), mg/dL	123 ± 37	123 ± 36	0.91
eGFR, mean (SD), mL/min/1.73 m^2^	65 ± 21	60 ± 24	0.018
History of hypertension, %	254 (71)	127 (71)	0.93
History of diabetes, %	66 (18)	35 (20)	0.73
Cardiovascular disease, %	167 (46)	101 (56)	0.026
Minor cardiovascular disease, %	107 (30)	74 (41)	0.007
Angina pectoris, %	52 (15)	37 (21)	0.069
TIA, %	31 (8.8)	24 (14)	0.085
Peripheral arterial disease, %	28 (7.8)	21 (12)	0.13
History of decompensated HF, %	34 (9.5)	24 (13)	0.17
Major cardiovascular disease, %	117 (32)	71 (40)	0.096
Myocardial infarction^a^, %	78 (22)	40 (22)	0.87
Stroke, %	23 (6.5)	23 (13)	0.013
PTCA or stent, %	32 (8.9)	14 (7.9)	0.70
Coronary surgery, %	27 (7.5)	8 (4.5)	0.18
Arterial surgery, %	15 (4.2)	12 (6.8)	0.20
Antihypertensive medication use, %	285 (79)	152 (85)	0.11
Cholesterol lowering medication use, %	121 (34)	50 (28)	0.18
Systolic BP, mean (SD), mmHg	142 ± 20	140 ± 21	0.25
Diastolic BP, mean (SD), mmHg	75 ± 9.5	75 ± 9.2	0.66

*Abbreviations*: *SD* standard deviation, *BMI* body mass index, *HDL-C* high-density lipoprotein cholesterol, *LDL-C* low-density lipoprotein cholesterol, *eGFR* estimated glomerular filtration rate (MDRD formula), *TIA* transient ischaemic attack, *HF* heart failure, *PTCA* percutaneous transluminal coronary angioplasty, *BP* blood pressure

^a^according to the GP or positive ECG (Minnesota Code 1–1 or 1–2 (excluding 1–2-8))

In Table 2 the association between traditional cardiovascular risk factors and all-cause and cardiovascular mortality was investigated in the total population (*n* = 566) and in patients without cardiovascular disease (*n* = 286) and patients with cardiovascular disease (*n* = 280) separately. In general, almost no classic cardiovascular risk factor was associated with mortality in the total population or in patients with or without cardiovascular disease. Only the level of HDL-C showed an inverse association with all-cause mortality only in the total population [adjusted hazard ratio (HR): 0.89, 95%CI: (0.81–0.98)]. A trend towards an inverse association with all-cause mortality was also observed for BMI and systolic blood pressure in patients without cardiovascular disease and between HDL-C and cardiovascular mortality in the total population.

**Table 2:** The association of classic cardiovascular risk factors and mortality in the total population and stratified by the presence of cardiovascular morbidity (*n* = 566)

	Total population (*n* = 566)		No cardiovascular disease (*n* = 286)		Cardiovascular disease (*n* = 280)	
	HR (95% CI)	*P* value	HR (95% CI)	*P* value	HR (95% CI)	*P* value
*All-cause mortality, n* ^a^ *(%)*	241 (43)		102 (36)		139 (50)	
Current/prior smoking	1.3 (0.94–1.8)	0.12	1.3 (0.76–2.2)	0.34	1.3 (0.84–1.9)	0.26
BMI, per kg/m^2^	0.99 (0.96–1.0)	0.45	0.96 (0.92–1.004)	0.076	1.01 (0.97–1.05)	0.57
Total cholesterol, per 10 mg/dL	0.99 (0.96–1.02)	0.48	0.999 (0.95–1.05)	0.95	1.001 (0.96–1.04)	0.96
HDL-C, per 10 mg/dL	0.89 (0.81–0.98)	0.020	0.94 (0.82–1.08)	0.37	0.90 (0.79–1.04)	0.14
LDL-C, per 10 mg/dL	1.0 (0.96–1.03)	0.77	0.996 (0.94–1.06)	0.90	1.02 (0.97–1.06)	0.54
History of hypertension	1.2 (0.91–1.6)	0.18	1.0 (0.66–1.7)	0.85	1.2 (0.80–1.7)	0.41
History of diabetes	1.1 (0.84–1.6)	0.40	1.2 (0.70–2.0)	0.54	1.1 (0.75–1.7)	0.59
Systolic BP, per 10 mmHg higher	0.97 (0.91–1.03)	0.34	0.90 (0.81–1.01)	0.062	1.01 (0.93–1.1)	0.78
Diastolic BP, per 10 mmHg higher	0.96 (0.84–1.1)	0.57	1.05 (0.85–1.3)	0.68	0.91 (0.77–1.08)	0.30
*Cardiovascular mortality, n* ^a^ *(%)*	104 (18)		40 (14)		64 (23)	
Current/prior smoking	1.2 (0.72–2.0)	0.47	0.61 (0.22–1.7)	0.34	1.5 (0.79–2.7)	0.23
BMI, per kg/m^2^	1.0 (0.96–1.05)	0.90	0.99 (0.93–1.06)	0.71	1.01 (0.96–1.07)	0.69
Total cholesterol, per 10 mg/dL	0.99 (0.94–1.03)	0.57	0.995 (0.92–1.07)	0.91	1.01 (0.95–1.07)	0.84
HDL-C, per 10 mg/dL	0.87 (0.76–1.01)	0.067	0.96 (0.77–1.2)	0.70	0.87 (0.70–1.07)	0.17
LDL-C, per 10 mg/dL	0.99 (0.94–1.05)	0.73	0.97 (0.89–1.07)	0.55	1.03 (0.96–1.1)	0.45
History of hypertension	1.2 (0.77–1.8)	0.45	0.81 (0.40–1.6)	0.54	1.3 (0.74–2.4)	0.35
History of diabetes	1.3 (0.81–2.1)	0.27	1.2 (0.52–2.7)	0.71	1.3 (0.75–2.4)	0.32
Systolic BP, per 10 mmHg higher	0.96 (0.87–1.06)	0.44	0.92 (0.78–1.08)	0.31	1.003 (0.88–1.1)	0.96
Diastolic BP, per 10 mmHg higher	0.95 (0.77–1.2)	0.59	1.1 (0.81–1.6)	0.47	0.87 (0.67–1.1)	0.26

All HR were adjusted for gender, age and level of education

*Abbreviations*: *HR* hazard ratio, *CI* confidence interval, *BMI* body mass index, *HDL-C* high density lipoprotein cholesterol, *LDL-C* low density lipoprotein cholesterol, *BP* blood pressure

^a^Observed number of deaths after 5 years of follow-up

**Fig. 1:**
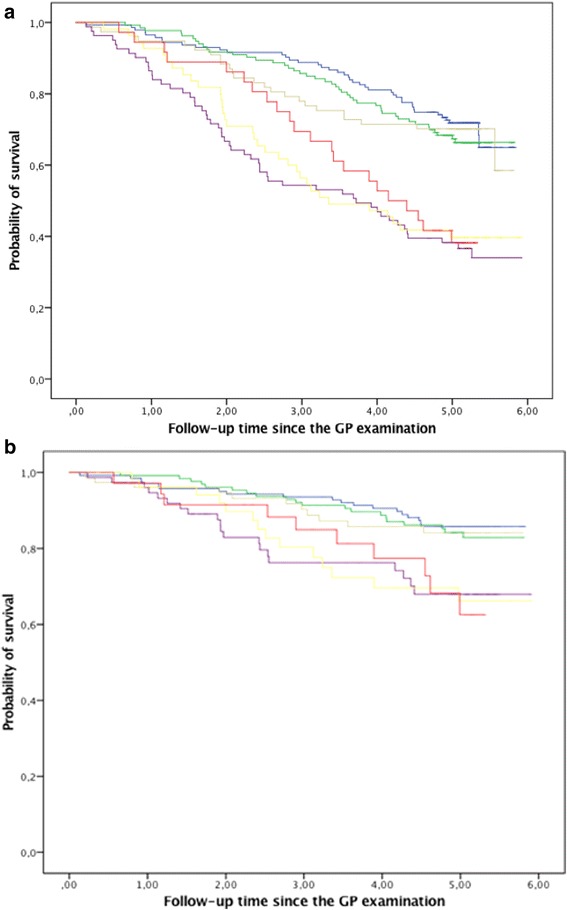
Survival from all-cause and cardiovascular mortality according to the presence of frailty and measured systolic blood pressure. **a** All-cause mortality. **b** Cardiovascular mortality. Figure legend: Robust and systolic BP <140 mmHg. Robust and systolic BP 140–160 mmHg.  Robust and systolic BP ≥160 mmHg.  Frail and systolic BP <140 mmHg.  Frail and systolic BP 140–160 mmHg.  Frail and systolic BP ≥160 mmHg

In Fig. 1, the Kaplan-Meier curves show the survival of patients according to their measured systolic blood pressure in combination with the presence of frailty (see Additional file 1: Figure S1, S2 and S3 for total cholesterol, HDL-C and diastolic blood pressure, respectively). The presence of frailty was able to identify patients at high risk for all-cause and cardiovascular mortality [adjusted HR: 2.5, 95%CI: (1.9–3.2) and adjusted HR: 2.2, 95%CI: (1.4–3.4), respectively]. However, within robust or frail patients, different categories of blood pressure and different tertiles of cholesterol were not able to further identify patients at risk for mortality (all log-rank tests, *P* > 0.05).

Table 3 shows the association of traditional cardiovascular risk factors and mortality according to the presence of frailty. In robust patients, HDL-C showed a protective effect for all-cause mortality, whereas the presence of hypertension and cardiovascular disease increased the risk. The multivariable model only retained the history of cardiovascular disease as a risk factor for all-cause mortality [adjusted HR: 1.4, 95%CI: (1.1–1.9)]. Furthermore, a history of cardiovascular disease increased the risk for cardiovascular mortality in robust persons. In frail individuals, no association with any of the traditional risk factors was found.

**Table 3:** Association of classic cardiovascular risk factors and mortality stratified by the presence of frailty (*n* = 541)

	Robust (*n* = 361)		Frail^a^ (*n* = 180)	
	HR (95% CI)	*P* value	HR (95% CI)	*P* value
*All-cause mortality, n* ^a^ *(%)*	114 (32)		110 (61)	
Current/prior smoking	1.3 (0.77–2.1)	0.35	1.2 (0.75–1.8)	0.50
BMI, per kg/m^2^	1.01 (0.96–1.05)	0.82	0.98 (0.94–1.01)	0.22
Total cholesterol, per 10 mg/dL	0.98 (0.94–1.02)	0.38	0.99 (0.94–1.03)	0.55
HDL-C, per 10 mg/dL	0.85 (0.74–0.99)	0.030	0.95 (0.84–1.08)	0.46
LDL-C, per 10 mg/dL	0.98 (0.93–1.04)	0.52	1.0 (0.95–1.05)	0.94
History of hypertension	1.6 (1.01–2.4)	0.047	1.2 (0.78–1.9)	0.39
History of diabetes	1.5 (0.97–2.3)	0.069	0.75 (0.44–1.3)	0.31
Systolic BP, per 10 mmHg higher	1.0 (0.91–1.1)	0.99	0.96 (0.88–1.1)	0.44
Diastolic BP, per 10 mmHg higher	1.05 (0.87–1.3)	0.60	0.89 (0.73–1.1)	0.27
Cardiovascular disease	1.7 (1.1–2.5)	0.011	1.07 (0.72–1.6)	0.74
*Cardiovascular mortality, n* ^a^ *(%)*	51 (14)		45 (25)	
Current/prior smoking	1.4 (0.62–3.0)	0.44	0.90 (0.45–1.8)	0.78
BMI, per kg/m^2^	0.98 (0.92–1.05)	0.60	1.0 (0.95–1.06)	0.94
Total cholesterol, per 10 mg/dL	1.0 (0.94–1.07)	0.98	0.96 (0.89–1.03)	0.24
HDL-C, per 10 mg/dL	0.86 (0.70–1.07)	0.17	0.90 (0.73–1.1)	0.30
LDL-C, per 10 mg/dL	1.01 (0.93–1.09)	0.87	0.96 (0.88–1.05)	0.39
History of hypertension	1.6 (0.82–3.2)	0.16	0.97 (0.51–1.8)	0.91
History of diabetes	1.5 (0.79–2.8)	0.21	0.93 (0.41–2.1)	0.87
Systolic BP, per 10 mmHg higher	0.96 (0.83–1.1)	0.61	0.99 (0.86–1.1)	0.88
Diastolic BP, per 10 mmHg higher	1.1 (0.82–1.5)	0.49	0.89 (0.65–1.2)	0.47
Cardiovascular disease	2.2 (1.2–3.9)	0.010	1.3 (0.71–2.5)	0.38

All presented HRs were only adjusted for gender, age and level of education, the HRs in the table were not adjusted for the presence of other cardiovascular risk factors

*Abbreviations*: *HR* hazard ratio, *CI* confidence interval, *BMI* body mass index, *HDL-C* high density lipoprotein cholesterol, *LDL-C* low density lipoprotein cholesterol, *BP* blood pressure

^a^Observed number of deaths after 5 years of follow-up

## Discussion

In this large, representative cohort of very old subjects aged 80 years and older, traditional cardiovascular risk factors did not show an association with all-cause or cardiovascular mortality. This pattern was observed both in subjects with and without cardiovascular disease. The presence of frailty, on the other hand, was able to identify patients at high risk for mortality. However, within the strata of robust and frail subjects, traditional cardiovascular risk factors were not able to further identify patients at risk of mortality. Only a history of cardiovascular disease showed a strong association with mortality in robust subjects.

The current study showed that classic cardiovascular risk factors were not associated with mortality in the oldest old. In the Leiden 85 Plus study, de Ruijter et al. [17] showed that classic risk factors included in the Framingham risk score could not identify patients at risk for cardiovascular mortality. This study was performed on a subpopulation of patients without a history of cardiovascular disease. Furthermore, in subjects with a history of cardiovascular disease in the same Leiden 85 Plus cohort, van Peet et al. [18] showed that traditional risk markers had little predictive value for recurrent cardiovascular events and cardiovascular mortality. Moreover, van Peet et al. showed that the history of cardiovascular disease is an important prognostic value in the oldest old [16]. The current study only found this association in robust subjects.

The current study confirmed the importance of frailty to identify patients at risk for mortality. However, the current study could not support the hypothesis that frailty could be used to further identify patients who might benefit more from cardiovascular risk management. This is in contrast with findings from the NHANES study where walking speed was used as a simple measure and as a proxy for frailty to identify elderly adults that were most at risk for adverse outcomes related to high blood pressure [10]. On the other hand, an extension of the HYVET trial did not show a difference in the effect of treatment between robust and frail individuals [19].

Although traditional cardiovascular risk factors lose their predictive value in the oldest old, new biomarkers, such as homocysteine and N-terminal pro-B-type natriuretic peptide (NT-proBNP), might be used to identify subjects at risk [17, 18]. As previously stated, cardiovascular risk management is a priority in this age group given the high burden of cardiovascular disease and high risk for cardiovascular morbidity and mortality. Therefore, the search for new strategies to identify patients at risk and who will benefit most from interventions in this heterogeneous group will be a research priority in the following years. On the other hand, the strong association between frailty and mortality further supports to follow a patient-based approach for future interventions instead of holding on to a disease-based approach in the oldest old.

The current study has several strengths. The BELFRAIL study is a large prospective cohort study representative of the general Belgian population aged 80 years and older. To our knowledge, this study was the first to investigate the association of traditional cardiovascular risk factors with mortality in robust and frail subjects, where frailty was operationalized based on three different validated models for frailty. However, a few limitations should be noted. First, using a combination of three different frailty models, all designed to cover a different latent construct of frailty, could lead to differential misdiagnosis. However, this approach was mainly used to identify ‘certainly robust’ subjects and identifying ‘possibly frail’ patients. Second, this was an observational study of a real world population, indicating that possible changes in medication intake after baseline might underlie the observed associations. Third, the comorbidities may have been underdiagnosed because they were reported by the general practitioner rather than being assessed. Fourth, only mortality was reported as outcome, although morbidity, such as stroke and heart failure, are also important outcomes in relation to traditional cardiovascular risk factors.

## Conclusions

In a large representative sample of very old subjects, traditional cardiovascular risk factors were not associated with mortality in subjects with and without cardiovascular disease. Frailty was shown to be a strong risk factor for mortality in this age group. However, frailty could not be used to identify further subjects who might benefit more from cardiovascular risk management. Only a history of cardiovascular disease was associated with mortality in robust people. Possibly, risk scores that include other risk factors, such as new biomarkers, are more suited to identify patients at risk in this age segment.

## Additional file

Additional file 1:
**Figure S1, S2** and **S3.** Survival from all-cause and cardiovascular mortality according to the presence of frailty and measured total cholesterol (Figure S1), HDL cholesterol (Figure S2) and diastolic blood pressure (Figure S3). (DOCX 124 kb)

## Abbreviations

ACE-I: Angiotensin-Converting Enzyme Inhibitor
ARB: Angiotensin II Receptor Blockers
BMI: Body Mass Index
CRA: Clinical Research Assistant
ECG: Electrocardiogram
eGFR: estimated Glomerular Filtration Rate
FEV1: Forced Expiratory Volume in one second
GDS: Geriatric Depression Scale
GFI: Groningen Frailty Indicator
GP: General practitioner
HDL-C: High-Density Lipoprotein Cholesterol
HDL-C: Low-Density Lipoprotein Cholesterol
HR: Hazard Ratio
LAPAQ: LASA Physical Activity Questionnaire
LASA: Longitudinal Aging Study Amsterdam
MDRD: Modification of Diet in Renal Disease
MMSE: Mini Mental State Examination
PTCA: Percutaneous transluminal coronary angioplasty
SD: Standard deviation
SOC: Sense Of Coherence
TIA: Transient Ischaemic Attack
