# Supplementary material for: Association between traditional cardiovascular risk factors and mortality in the oldest old: untangling the role of frailty
Source: BMC Geriatr. 2017 Oct 12;17:234. doi: 10.1186/s12877-017-0626-x (PMC5639737; doi:10.1186/s12877-017-0626-x)
Supplement: Additional file 1: — Figure S1, S2 and S3. Survival from all-cause and cardiovascular mortality according to the presence of frailty and measured total cholesterol (Figure S1), HDL cholesterol (Figure S2) and diastolic blood pressure (Figure S3). (DOCX 124 kb) [file 12877_2017_626_MOESM1_ESM.docx]

Additional file

| **Figure 1, 2 and 3.** | |
| --- | --- |
| **All-cause mortality** | **Cardiovascular mortality** |
| ***Total cholesterol (Figure 1)*** |  |
| 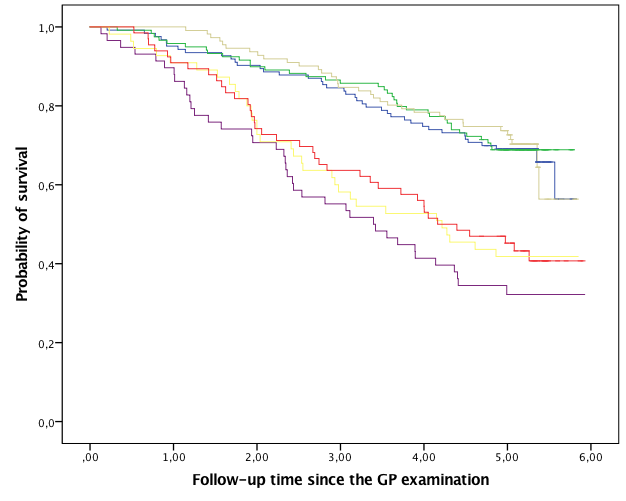 | 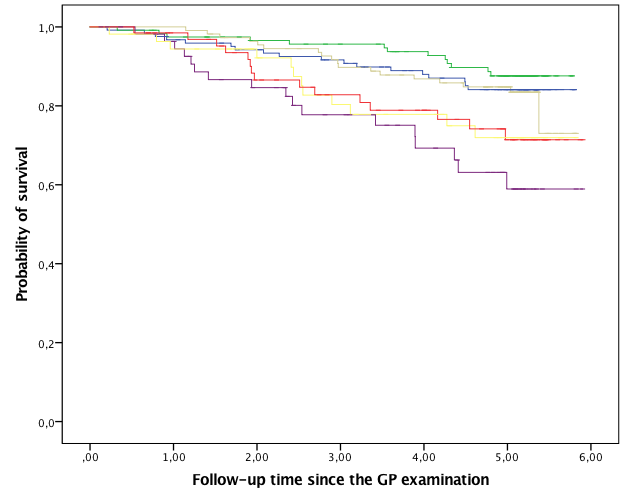 |
| **____** Robust and lowest tertile of total cholesterol (<179mg/dL)  **____** Robust and middle tertile of total cholesterol (179 - 218mg/dL)  **____** Robust and highest tertile of total cholesterol (>218mg/dL)  **____** Frail and lowest tertile of total cholesterol (<179mg/dL)  **____** Frail and middle tertile of total cholesterol (179 - 218mg/dL)  **____** Frail and highest tertile of total cholesterol (>218mg/dL) | |
| ***HDL cholesterol (Figure 2)*** |  |
| 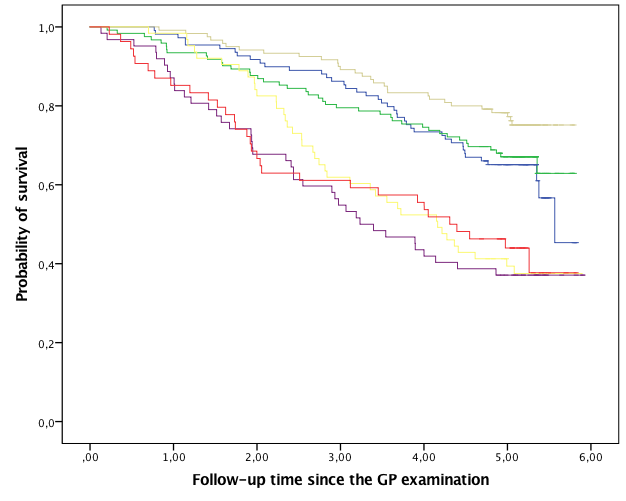 | 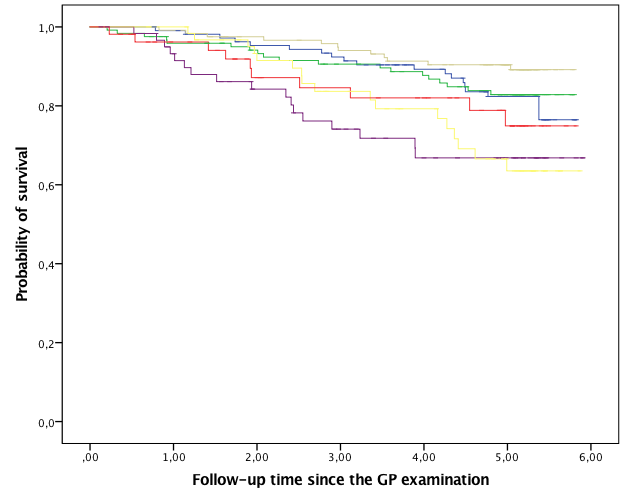 |
| **____** Robust and lowest tertile of HDL-C (<48mg/dL)  **____** Robust and middle tertile of HDL-C (48 - 60mg/dL)  **____** Robust and highest tertile of HDL-C (>60mg/dL)  **____** Frail and lowest tertile of HDL-C (<48mg/dL)  **____** Frail and middle tertile of HDL-C (48 - 60mg/dL)  **____** Frail and highest tertile of HDL-C (>60mg/dL) | |
| ***Diastolic blood pressure (Figure 3)*** |  |
| 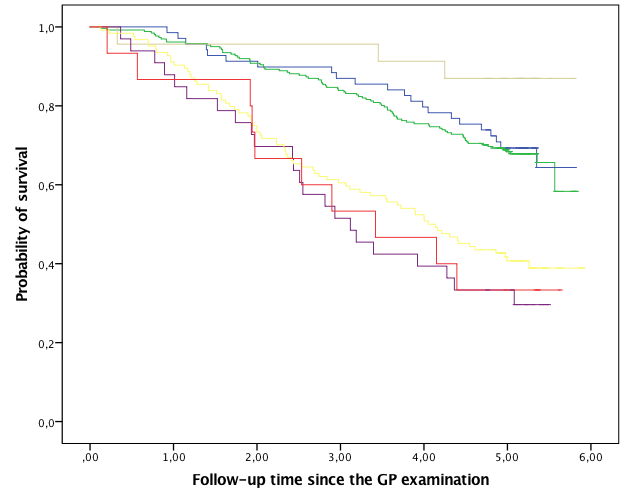 | 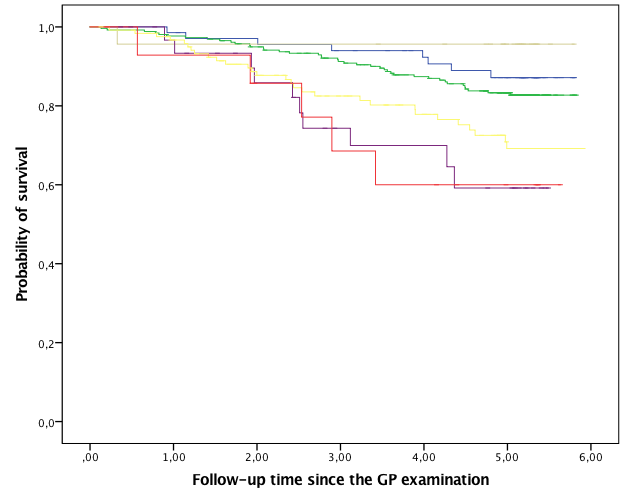 |
| **____** Robust and diastolic BP <70mmHg  **____** Robust and diastolic BP 70-90mmHg  **____** Robust and diastolic BP ≥90mmHg  **____** Frail and diastolic BP <70mmHg  **____** Frail and diastolic BP 70-90mmHg  **____** Frail and diastolic BP ≥90mmHg | |
